# F128. THE AGE OF ONSET OF SCHIZOPHRENIA SPECTRUM DISORDERS

**DOI:** 10.1093/schbul/sby017.659

**Published:** 2018-04-01

**Authors:** Jouko Miettunen, Johanna Immonen, John McGrath, Matti Isohanni, Erika Jääskeläinen

**Affiliations:** 1 University of Oulu; 2 University of Queensland, Queensland Brain Institute

## Abstract

**Background:**

This study characterizes the age of onset of schizophrenia spectrum disorders and summarizes findings regarding a range of clinical and social outcomes, cognition, brain structure, and mortality.

**Methods:**

The review is based on series of systematic and nonsystematic literature searches. We included original articles and systematic reviews looking associations between age of onset and incidence, risk factors, suicides, brain structure and cognition.

**Results:**

The peak age of onset for schizophrenia spectrum disorders is between 20 to 29 years, in where the incidence estimate was among males 4.15 and among females 1.71 per 10,000 person-years. Male gender has been linked with earlier onset age, although among those with family history and cannabis use corresponding gender difference do not exist. Early onset schizophrenia has been linked e.g. with higher familial risk, poor premorbid social adjustment and cannabis use. In adult samples, earlier age of onset associated with worse outcome, regarding hospitalisations, negative symptoms, relapses, social and occupational functioning, and global outcome. Also in childhood and adolescence schizophrenia, earlier onset has been linked with more severe outcomes. Early age of onset has been linked also with larger cognitive deficits and brain alterations. In the few existing studies, later AOO has been linked with a higher suicide rate. In all, the current study found various differences between patients with different age of onset. However, the studies on age of onset are relative heterogeneous on methodology and have given varying results. More good quality studies are needed including patients without restriction due to the onset age.

**Discussion:**

Age of onset is an important characteristic of schizophrenia that could help when examining the origin, genetic mechanism and care of schizophrenia. Understanding factors that influence age of onset in schizophrenia may offer clues to prevent or delay the onset of this debilitating group of disorders.

